# First Trimester Evaluation of Maternal Visceral Fat and Its Relationship with Adverse Pregnancy Outcomes

**DOI:** 10.3390/biology12020144

**Published:** 2023-01-17

**Authors:** Francisco Brenes-Martín, Victoria Melero-Jiménez, Miguel Ángel López-Guerrero, María Mercedes Calero-Ruiz, Luis Vázquez-Fonseca, Jessica Ábalos-Martínez, Rocío Quintero-Prado, Rafael Torrejón, Francisco Visiedo, Fernando Bugatto

**Affiliations:** 1 Division of Maternal-Fetal Medicine, Obstetrics and Gynecology Department, Puerta del Mar University Hospital, 11009 Cádiz, Spain; 2 Inflammation and Metabolic Syndrome in Pregnancy Group (CO25), Biomedical Research and Innovation Institute of Cádiz (INiBICA), 11009 Cádiz, Spain; 3 Clinical Laboratory Department, Puerta del Mar University Hospital, 11009 Cádiz, Spain; 4 Area of Obstetrics and Gynaecology, Department of Child and Mother Health and Radiology, School of Medicine, University of Cádiz, 11009 Cádiz, Spain

**Keywords:** visceral fat, subcutaneous fat, adverse pregnancy outcomes, adiposity, maternal obesity, pregnancy

## Abstract

**Simple Summary:**

Maternal adipose tissue grows during pregnancy to secure the fetus’s nutritional supply, and too much visceral adipose tissue at the start of pregnancy can increase metabolic risk and gestational problems. The distribution of fat, and more particularly the rise in visceral fat or central obesity, has been found to be more closely linked to the onset of cardiovascular disease and metabolic syndrome than obesity itself. Our goal is to examine the association between the thickness of the mother’s visceral fat, as determined by a first trimester ultrasound exam, and the risk of poor pregnancy outcomes. We observed that women who experienced complications during pregnancy had greater levels of maternal visceral fat, especially gestational diabetes, which was linked to metabolic risk factors including insulin resistance and arterial blood pressure. This fact may imply that the risk of complications would increase more when the distribution of visceral fat (associated with metabolic risk) is greater than expected for a given degree of obesity/body mass index.

**Abstract:**

Obese women are more likely to experience pregnancy complications. The distribution of fat, and more particularly the rise in visceral fat, is well established to be more closely linked to the onset of cardiovascular disease and metabolic syndrome than obesity itself. We aim to examine the relationship between maternal visceral fat assessment in the first trimester and the appearance of adverse pregnancy outcomes. A prospective cohort study including 416 pregnant women was conducted. During the first trimester scan (11–13 + 6 weeks), all individuals had their visceral fat and subcutaneous thicknesses measured by ultrasonography. Blood samples were obtained, and maternal demographics and clinical information were documented. After delivery, the obstetric outcomes were evaluated. We contrasted two groups: one with healthy pregnancies and the other with adverse pregnancy outcomes (APO), defined as the development of at least one of the following complications: gestational diabetes mellitus, hypertensive disorders of pregnancy, abnormal fetal growth, preterm delivery or preterm premature rupture of membranes. Median maternal age was 33 and 34 years old for the uncomplicated and adverse pregnancy outcomes groups, respectively. We found that women with adverse pregnancy outcomes had higher VFT (median 30 vs. 26.5 mm, *p* = 0.001) and SFT (median 18.9 vs. 17.1 mm, *p* = 0.03). However, the visceral/subcutaneous fat ratio was not statistically different between groups. Finally, we performed a subanalysis for metabolic and placental vascular dysfunction complications. After performing a multivariate logistic regression analysis adjusted for maternal age, smoking, and mean arterial pressure, both the VFT (aOR 1.03, *p* < 0.001) and the ratio of visceral/subcutaneous fat (aOR 1.37, *p* = 0.04) were significantly associated with the development of adverse pregnancy outcomes; however, the associations of VFT and the VFT-to-SFT ratio were higher for the occurrence of gestational diabetes (aOR 1.07, *p* < 0.001; aOR 2.09, *p* = 0.001; respectively) and showed no relationships with placental complications. When conducting a first-trimester ultrasound assessment, sonographers may measure VFT without additional time or cost involved. Identification of pregnant women with increased VFT (>37 mm) may benefit from a close follow-up, especially for the development of gestational diabetes, independent of BMI.

## 1. Introduction

The incidence of obesity among reproductive-age women has increased in recent decades due to rising sedentary and obesity rates globally [1] and complicates approximately one-fifth of pregnancies [2]. Due to the prevalence of obesity in women, the consequences for pregnancy are frequently underappreciated, disregarded, or ignored. This is because there are no particular evidence-based treatment options available [3]. Obesity is presently viewed as a chronic inflammatory condition having detrimental effects on health and is linked to a number of metabolic and cardiovascular conditions, such as hypertension, arteriosclerosis, glucose intolerance, dyslipidemia, and insulin resistance. Although body mass index (BMI), which is determined by dividing a person’s weight in kilograms by their height in meters squared (Kg/m^2^), is a common way to categorize obesity, it has been demonstrated that the distribution of fat, and more specifically the accumulation of visceral fat or central obesity, is the aspect of obesity that is most closely linked to the onset of cardiovascular disease and metabolic syndrome [4,5,6]. Antepartum, intrapartum, and postpartum complications associated with obesity during pregnancy include miscarriage, venous thromboembolism, gestational diabetes mellitus, preeclampsia, cardiac dysfunction, sleep apnea, nonalcoholic fatty liver disease, mental disorders, instrumental vaginal delivery, cesarean section, wound infections, postpartum hemorrhage, and complications from anesthesia [3,7].

Numerous studies have also shown that this medical condition may cause fetal and neonatal complications, such as congenital anomalies, stillbirth, macrosomia, prematurity, shoulder dystocia, admission to neonatal intensive care unit, neonatal death, and a long-term increase in the offspring’s cardiometabolic risk [7,8,9,10]. Additionally, there is an increase in adipose tissue throughout pregnancy to maintain the fetus’s nutritional supply, and too much visceral adipose tissue at the beginning of pregnancy may raise the risk of metabolic disorders and gestational complications. Visceral fat thickness (VFT) can be easily measured in the first trimester scan and correlates better with metabolic risk factors than BMI (diastolic blood pressure, blood glucose, insulinemia, insulin sensitivity, triglycerides, HDL cholesterol, and total cholesterol/HDL cholesterol ratio) [11]. The thickness of visceral fat has also been associated with the appearance of metabolic syndrome and gestational diabetes and has been proposed as a possible screening method for gestational diabetes [12,13,14].

The first trimester of pregnancy is an essential time frame in the current model of prenatal care. In recent times, due to the development of ultrasound and new biochemical biomarkers, we have assisted the birth of a model for a new pyramid of prenatal care based on the 11 to 13 weeks’ assessment [15]. Using fetal and maternal characteristics, a first-trimester pregnancy evaluation can help determine the likelihood of complications that clinically manifest later in pregnancy. Individualized prenatal treatment is made feasible by this assessment [16].

Given that VFT is associated with the development of metabolic syndrome and that pregnancy can be thought of as a brief excursion into the syndrome due to the environment it creates [17,18,19], our goal is to investigate the connection between the assessment of maternal visceral fat during the first trimester and the emergence of adverse pregnancy outcomes.

## 2. Materials and Methods

### 2.1. Study Population

A prospective cohort study was conducted in a tertiary referral university center during the years 2021–2022. Just after the first trimester scan (11 to 14 weeks), we enrolled 416 healthy women with a singleton pregnancy from our antenatal clinics, and they gave their complete, informed consent to take part in the study. Crown-rump length (CRL), fetal anatomy, mean uterine arteries Pulsatility Index (mUtA-PI) and nuchal translucency were evaluated and measured prior to the study, following the ISUOG Practice Guidelines [20]. Abnormal karyotype, fetal malformations, multiple gestation, or maternal diseases such as infections, diabetes, or hypertension were exclusion criteria. Additionally, systolic and diastolic blood pressure (SBP, DBP) were checked in each arm in triplicate, and mean arterial pressure value was recorded (MAP = [SBP + (2 × DBP)]/3). Height, and pregestational weight were recorded at prenatal visit, and prepregnancy BMI was calculated. Median maternal age was 33 and 34 years old for the uncomplicated and adverse pregnancy outcomes groups, respectively.

### 2.2. Ultrasound Measurements

**Figure 1:**
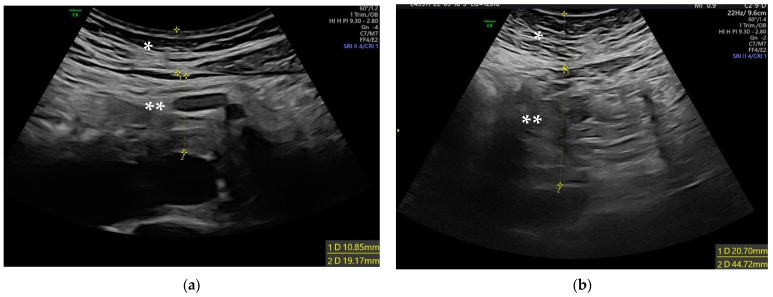
Representative abdominal first trimester ultrasound measurement of maximum subcutaneous fat thickness (*) and visceral fat thickness (**) in lean (**a**) and obese women (**b**).

A 2–9 MHz abdominal transducer on a Voluson^TM^ E8 ultrasound system (XDclear^TM^ probe C2-9-D, GE Healthcare, Milwaukee, Wisconsin, USA) was used to perform ultrasound scans. To calculate the maximum subcutaneous fat thickness (SFT) and VFT, transverse scanning was used. Both measurements were made 1 cm above the umbilicus, near the midline of the abdomen (Figure 1) [11]. To prevent being influenced by respiratory activity or abdominal wall tightness, all frozen pictures were taken immediately after expiration. Without applying excessive pressure that would have altered the thickness and shape of the body layer, the transducer was placed on the body’s surface. The space between the internal layer of skin and the exterior face of the rectoabdominal muscle was named the SFT. VFT was defined as the distance, measured perpendicular to the aorta, between the internal layer of the transversalis fascia and the anterior wall. The ratio of VFT-to-SFT was determined.

### 2.3. Biochemical Analysis

A prior venous blood plasma sample used for the first trimester combined screening test for Down syndrome was taken at 10 weeks of pregnancy and stored at −20 °C until samples from women participating in the study were analyzed. Fasting glucose was determined in venous blood using the Modular DPD biochemical system (Roche Diagnostics, Basel, Switzerland). Glucose, insulin and lipid profiles, including total cholesterol, triglycerides (TG), LDL cholesterol (LDL-c) and HDL cholesterol (HDL-c) were quantified in the Modular DPD biochemical auto-analyzer using enzymatic colorimetry. Measurements of sFlt-1 and PlGF were performed on single serum samples using the Elecsys sFlt-1 and PlGF assays (Roche Diagnostics), an automated electrochemiluminescence immunoassay method [21]. A high-sensitivity CRP assay (Roche Diagnostics, Mannheim, Germany) was used for the quantification of C-reactive protein (CRP) according to the manufacturer’s instructions.

### 2.4. Diagnosis of Adverse Pregnancy Outcomes

The onset of at least one of the following pregnancy complications: gestational diabetes mellitus (GDM), hypertensive disorders of pregnancy, abnormal fetal growth (small for gestational age, intrauterine growth restriction-IUGR), preterm delivery, or preterm premature rupture of membranes before 37 weeks, was considered to result in an adverse pregnancy outcome [22]. GDM was determined using the two-step approach and 100 g-OGTT with two or more values exceeding 105, 190, 165, and 145 mg/dL after 0, 60, 120, and 180 min in accordance with the National Diabetes Data Group (NDDG) standards [23]. Birth weight centiles were obtained using customized birth weight criteria for a Spanish population, taking into account the fetal sex and gestational age at delivery [24]. Small for gestational age (SGA) was defined by a birth weight below the 10th customized weight-for-gestation centile and IUGR if abnormal Doppler was present [25]. Hypertensive disorder of pregnancy was diagnosed based on the following criteria: healthy, normotensive women with a blood pressure recording of 140/90 mmHg on ≥2 occasions for at least 4 h intervals after week 20 of pregnancy without proteinuria or altered angiogenic ratio. Preeclampsia was considered on the basis of the same blood pressure criteria and presence of proteinuria (≥300 mg per 24 h that was assessed by 24 h urine collections) and/or altered angiogenic ratio (sFlt-1/PlGF).

It was decided to homogenize pregnancy complications into two categories. The onset of gestational diabetes mellitus was designated as the first subgroup of metabolic problems. Presence of one of the following conditions was used to identify the second subgroup of related placental vascular dysfunction complications: hypertensive disorders of pregnancy/preeclampsia and/or abnormal fetal growth (SGA, IUGR).

### 2.5. Statistical Analysis

Statistical analysis was performed using the SPSS 25.0 for Windows (SPSS, Inc., Chicago, IL, USA) computer statistics program. Distributions were checked with a histogram and the Kolmogorov–Smirnov test. Comparisons between 2 groups were performed by using either the Student’s *t*-test or the Mann–Whitney U test (2-tailed) according to the normal or nonnormal distributions of the variables. The relationships between variables were analyzed using the Pearson or Spearman correlation coefficient when using parametric or nonparametric data, respectively. Statistical significance was set at the 95% level (*p* < 0.05). Multivariate analysis was performed by logistic regression to determine the effect of first trimester ultrasound parameters (maternal subcutaneous and visceral fat thickness) on the composite of adverse pregnancy outcomes. ROC curve was used for the evaluation of the area under the curve (AUC) as well as for the sensitivity and false-positive rate for different cutoffs of ultrasound parameters. The research’s design limitations stem from the fact that it was a cohort study conducted at a single center without randomization and without being blind to any of the components.

## 3. Results

### 3.1. Clinical and Anthropometric Characteristics

**Figure 2:**
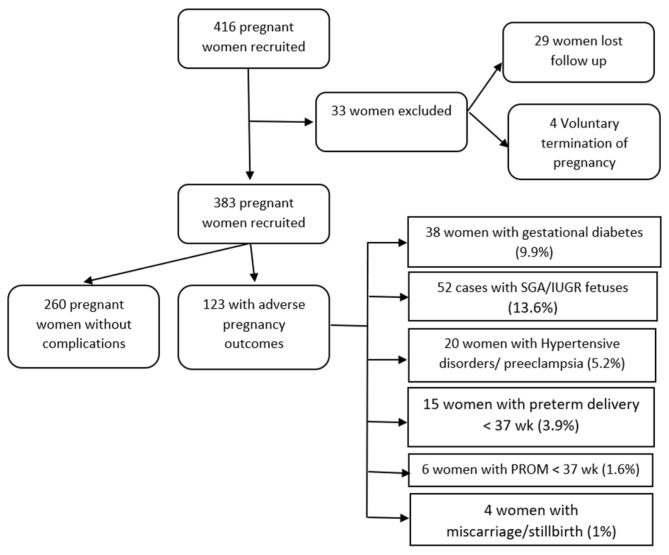
Study flow chart: algorithm for the identification and classification of eligible women. SGA/IUGR: small for gestational age/intrauterine growth restriction; and PROM: premature rupture of membranes.

Demographic and clinical characteristics of the 383 studied pregnant women finally studied are shown in Figure 2 and Table 1 according to the development of adverse pregnancy outcomes. The most frequent adverse outcomes occurring throughout pregnancy were abnormal fetal growth (n = 52, 13.6%), gestational diabetes (n = 38, 9.9%) and hypertensive disorders of pregnancy (n = 20, 5.2%), followed by preterm delivery before 37 weeks (n = 15, 3.9%), premature rupture of membranes before 37 weeks (n = 6, 1.6%) and stillbirth (n = 4, 1%). There were statistically significant differences between the group without complications (n = 260) and the group with adverse pregnancy outcomes (APO, n = 123) in terms of pregestational weight and BMI, gestational age at delivery, newborn centile and weight, smoking, systolic and diastolic blood pressure and mean arterial pressure.

### 3.2. Biochemical Analysis and Clinical Parameters Assessed

Biochemical parameters in maternal blood and ultrasound parameter measurements are listed in Table 2 according to the development of adverse pregnancy outcomes. APO group showed higher triglycerides (95 vs. 77 mg/dL, *p* = 0.001), LDL cholesterol (63.9 vs. 56.1 mg/dL, *p* = 0.02) and CRP levels (3.6 vs. 2.6 mg/L, *p* = 0.04) and lower sFlt-1 (1379 vs. 1579 pg/mL, *p* = 0.005) and sFlt-1/PlGF ratio (43.2 vs. 48.4, *p* = 0.03).

### 3.3. Ultrasound Measurements and Their Relationship with Biochemical and Clinical Parameters

**Figure 3:**
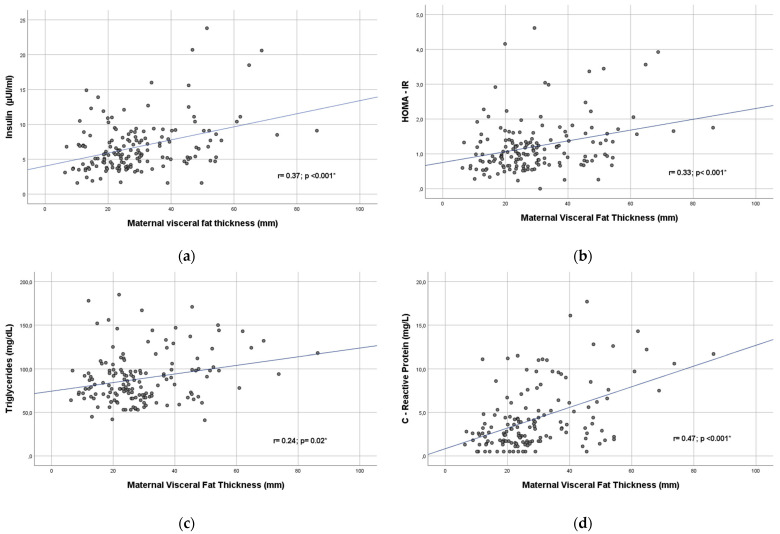

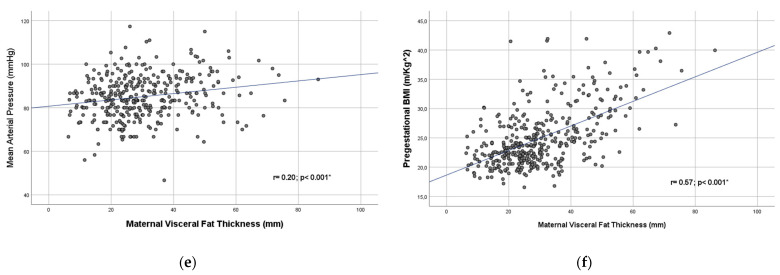
Scatterplot of maternal insulin (**a**), Insulin Resistant Index (HOMA-IR) (**b**), triglycerides (**c**), C-reactive protein (**d**), mean arterial pressure (**e**) and pregestational BMI (**f**) in relation to first trimester maternal visceral fat thickness. (*) Statistical significance *p* < 0.05.

Regarding ultrasound parameters, the APO group showed higher VFT (30 vs. 26 mm, *p* = 0.001) and SFT (18.9 vs. 17.1 mm, *p* = 0.03) than the group without pregnancy complications. There were no statistically significant differences in VFT/SFT ratio nor mean uterine arteries PI between groups. Maternal visceral and subcutaneous fat thickness showed significant correlations (Table 3 and Figure 3) with insulin (r = 0.37, *p* < 0.001; r = 0.30, *p* < 0.001, respectively) and HOMA-IR index (r = 0.33, *p* < 0.001; r = 0.34, *p* < 0.001, respectively) as well as with CRP (r = 0.47, *p* = 0.001; r = 0.28, *p* < 0.001, respectively), MAP (r = 0.20, *p* < 0.001; r = 0.27, *p* < 0.001, respectively) and BMI (r = 0.47; *p* < 0.001; r = 0.65; *p* < 0.001). Maternal visceral fat thickness showed a significant correlation with triglycerides (r = 0.24; *p* = 0.02). A negative significant correlation between subcutaneous fat, and mean uterine arteries PI (r = −0.16; *p* = 0.01) was also found.

Regarding ultrasound parameters, the group with metabolic complications showed higher VFT (46.8 vs. 27 mm, *p* < 0.001), SFT (21.1 vs. 17.2 mm, *p* < 0.001), and VFT-to-SFT ratio (1.78 vs. 1.55) and lower mean uterine arteries PI (1.4 vs. 1.6) than the group without pregnancy complications. There were no statistically significant differences in ultrasound parameters between groups with and without placental vascular dysfunction complications.

### 3.4. Logistic Regression Analysis

Multivariate analysis was performed by logistic regression to determine the effect of first trimester ultrasound parameters (maternal subcutaneous and visceral fat thickness) on the composite of adverse pregnancy outcomes with models adjusted for maternal age, tobacco use and mean arterial pressure (Table 4). On multivariate analysis, the adjusted OR of maternal visceral fat thickness on the presence of adverse perinatal outcome was 1.03 (95% CI, 1.01–1.04; *p* < 0.001). The adjusted OR of the ratio of VFT to SFT was also significant on the development of APO with a value of 1.37 (95% CI, 1.01–1.84; *p* = 0.04). However, regarding multivariate analysis, the adjusted OR of maternal pregestational BMI on the presence of adverse perinatal outcome was 1.03 and did not show statistical significance (95% CI, 0.98–1.08; *p* = 0.2). Finally, multivariate analysis was performed for the subgroups of metabolic and placental vascular dysfunction complications. The adjusted OR of all variables on metabolic complications of pregnancy showed statistical significance, showing that the ratio of VFT to SFT has higher association with an aOR of 2.09 (95% CI, 1.35–3.25). Nevertheless, none of studied variables showed association with the presence of vasculo-placental complications.

### 3.5. Receiver Operating Characteristic (ROC) Curves Analysis

**Figure 4:**
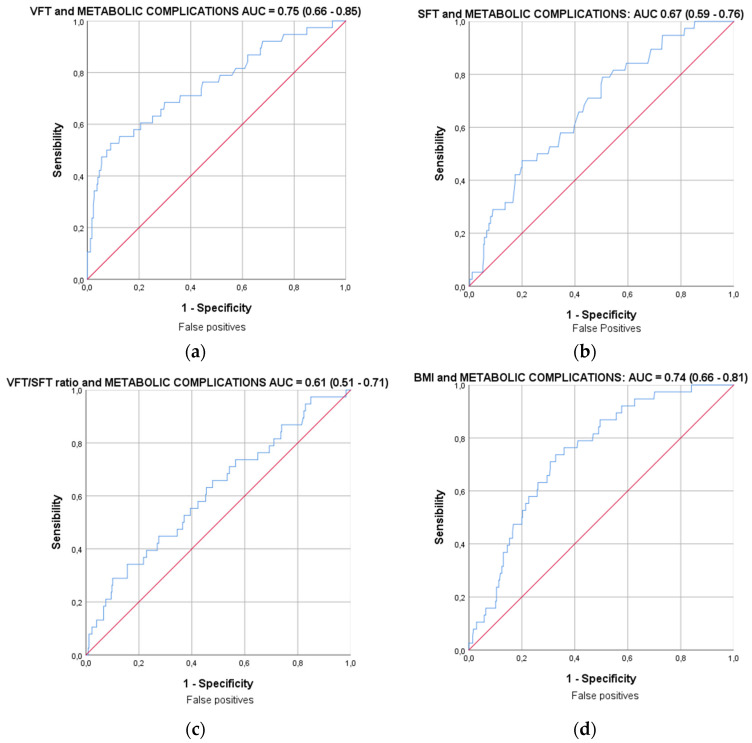
Receiver operating characteristic (ROC) curves for discrimination of the development of metabolic complications by means of VFT (**a**), SFT (**b**), VFT-to-SFT ratio (**c**) and BMI (**d**).

To determine the diagnostic performance of studied variables on the development of pregnancy complications, ROC curves were constructed. This analysis showed a poor diagnostic performance of ultrasound measurements for the composite of adverse pregnancy outcomes. The AUC for VFT was 0.60 (95% CI, 0.53–0.66), for SVF was 0.56 (CI 95%, 0.505–0.62), for the VFT-to-SFT ratio was 0.53 (95% CI, 0.47–0.59) and for BMI was 0.59 (95% CI, 0.53–0.65). However, the diagnostic performance on the occurrence of metabolic disorders (gestational diabetes mellitus) was better. The sole measurement of VFT performed best in the diagnosis of metabolic complications, compared to the VFT-to-SFT ratio or to the BMI. The AUC for the VFT was 0.75 (95% CI, 0.66–0.85). The AUC for SFT was 0.67 (95% CI, 0.59–0.76). The AUC for the VFT-to-SFT ratio was 0.61 (95% CI, 0.51–0.66). The AUC for BMI was 0.74 (95% CI, 0.66–0.81) (Figure 4). In the subgroup of metabolic complications, a cutoff for VFT of 37 mm results in sensitivity of 60% and a specificity of 80%, and a cutoff for BMI of 27.6 kg/m^2^ results in sensitivity of 50% and a specificity of 80%.

## 4. Discussion

The present study evaluated pregnant women in the first trimester of pregnancy by means of clinical, ultrasound and analytical parameters and subsequently followed up the course of the pregnancy. We measured SFT and VFT and analyzed their relationships with the development of gestational complications and the studied parameters.

The APO group presented higher maternal age, weight, pregestational BMI as well as higher values of SAP, DAP and MAP, since they all are known risk factors for the development of gestational complications. In addition, the birthweights and percentiles of the newborns were lower in the group with complications, since this was one of the criteria used to designate the group with complications (SGA/IUGR fetuses). The APO group presented a greater thickness of both maternal visceral and subcutaneous fat, and in line with the results obtained, a recent meta-analysis showed significantly increased odds of pregnancy complications with early pregnancy measures of adiposity, such as higher waist circumference or waist to hip ratio [26]. The group that developed gestational diabetes showed higher VFT, SFT and VFT-to-SFT ratio than the group without metabolic complications, since all maternal adiposity measurements are related to increased insulin resistance [18].

On the other hand, the group with gestational complications also presented higher levels of proinflammatory markers (CRP) and triglycerides. A proinflammatory environment is already more prevalent during pregnancy, and multiple studies have shown that it is linked to diseases including diabetes, preeclampsia, premature labor, and obesity [27,28,29,30]. Lower levels of sFlt-1 and the angiogenic sFlt-1/PlGF ratio were seen in the APO group. It might be explained by the fact that this is a marker of endothelial damage and preeclampsia and that a wider range of gestational complications (gestational diabetes, preterm delivery, IUGR, and so on) have been taken into account despite the fact that they are not typically associated with this marker. In this context, a recent systematic review concluded that there was no convincing evidence linking first-trimester sFlt-1 levels to unfavorable pregnancy outcomes [31].

We found statistically significant correlations between maternal visceral fat in the first trimester of pregnancy and several analytical and clinical parameters related to metabolic risk, including insulin and HOMA-IR index, triglycerides, blood pressure and BMI. These results are similar to those previously found by other authors [6] and emphasize the relevant paper played by visceral fat as a metabolic risk factor. After performing multivariate logistic regression adjusted for maternal age, smoking and MAP, both visceral fat thickness (aOR 1.03, 95% CI 1.01–1.04; *p*< 0.001) and visceral/subcutaneous fat ratio (aOR 1.37, 95% CI 1.01–1.85; *p* = 0.04) were significantly associated with the development of gestational complications, while subcutaneous fat thickness did not show this association. However, all ultrasound adiposity markers, including VFT, SFT and the VFT-to-SFT ratio showed significant associations with the development of gestational diabetes (aOR 1.07, *p* < 0.001; aOR 1.04, *p* = 0.03; and aOR 2.09, *p* = 0.001, respectively). This can be explained by the fact that ultrasound adiposity markers, and specially the visceral fat, are associated with metabolic risk factors [18]. Accordingly, some authors have proposed the evaluation of ultrasound measurements of abdominal fat for the prediction of gestational diabetes [32,33,34].

The result obtained can be interpreted by establishing that each 1 mm increase in maternal visceral fat represents a 7% increase in the risk of developing gestational diabetes. This increase in risk does not seem clinically relevant, but this is a measurement in millimeters and that, for example, for 10 or 20 mm, the increase in risk is 70% or 140% with respect to a woman with less visceral fat. The development of placental problems was not associated with any of the ultrasonography adiposity indicators. Comparable to this finding, in a previous study aimed at assessing the ability of isolated maternal periumbilical and epigastric fat measurements during pregnancy to predict hypertensive outcomes, these measurements were not able to predict preeclampsia [35].

The VFT-to-SFT ratio has shown an association with pregnancy complications with an aOR of 1.37 and a higher association with gestational diabetes (aOR 2.09), which would imply that an increase in the ratio of one unit would increase the risk of complications by 37% and more than double the risk of gestational diabetes. By contrast, BMI did not show association with adverse pregnancy outcomes and showed a lower aOR of 1.11 with gestational diabetes. These data suggest that the ratio of visceral to subcutaneous fat, rather than a single assessment of visceral fat thickness, may be more indicative of the risk of obstetric complications. This fact may imply that the risk of complications would increase more when the distribution of visceral fat (associated with metabolic risk) is greater than expected for a given degree of BMI or obesity (more related to subcutaneous fat). Several scientists have suggested that body fat distribution should be taken into account rather than raw body weight when studying obesity as a risk factor [36].

Finally, in the same manner, the ROC analysis revealed that the sole measurement of VFT performed best in the diagnosis of metabolic complications, compared to the BMI, improving by 10% the sensibility with the same rate of false positives (20%) with a cutoff of 37 mm.

## 5. Conclusions

We found that increased maternal visceral fat thickness is associated with adverse pregnancy outcomes and specially with gestational diabetes. This fact may imply that the risk of metabolic complications would increase more when the distribution of visceral fat (associated with metabolic risk) is greater than expected for a given degree of obesity/body mass index.

When conducting a first-trimester ultrasound assessment, sonographers may measure maternal VFT or VFT/SFT without additional time or cost involved. Based on our research, ultrasound adiposity measurements are more accurate than BMI at predicting global and metabolic risks during pregnancy and in order to provide care to pregnant women who are most in need.

## Figures and Tables

**Table 1:** Maternal demographics and clinical characteristics of the study’s participants. SBP: systolic blood pressure; DBP: diastolic blood pressure; MAP: mean arterial pressure.

Variable	Uncomplicated Group (n = 260)	Adverse Pregnancy Outcomes Group (n = 123)	*p* Value
Maternal age (years)	33 (29–36)	34 (30–37)	*p* = 0.05 *
Height (m)	1.63 ± 0.06	1.63 ± 0.06	*p* = 0.93
Pregestational weight (Kg)	62 (57–70.7)	66.3 (58–76)	*p* = 0.005 *
Pregestational BMI (Kg/m^2^)	23.3 (21.1–26.5)	24.9 (21.9–28.5)	*p* = 0.003 *
Nulliparous (n, %)	145 (56%)	67 (54.5%)	*p* = 0.34
Weight gain (kg)	11 (8.5–15)	11.5 (7.5–14)	*p* = 0.56
Gestational age at birth (weeks)	40 (39–40)	39 (37–40)	*p* = 0.001 *
Newborn male sex (n, %)	129 (50.4%)	58 (49.6%)	*p* = 0.88
Newborn weight (Kg)	3363 ± 365	2957 ± 668	*p* = 0.001 *
Newborn centile	47 (30–65.7)	23 (6–67)	*p* = 0.001 *
SBP (mm Hg)	113 (105–122)	120 (110–128)	*p* = 0.001 *
DBP (mmHg)	70 (60–75)	72 (65.5–80)	*p* = 0.001 *
MAP (mmHg)	83.5 ± 9.3	88.1 ± 10	*p* = 0.001 *
Smoking (N, %)	22 (8.7%)	20 (16.3%)	*p* = 0.02 *

(*) Statistical significance *p* < 0.05.

**Table 2:** First trimester ultrasound parameters and laboratory variables, according to presence of adverse pregnancy outcomes. CRP: C-reactive protein; PlGF: placental growth factor; sFlt-1: soluble tyrosine kinase 1 similar to fms; SFT: subcutaneous fat thickness; VFT: visceral fat thickness; PI: Pulsatility Index.

Variable	Uncomplicated Group (n = 260)	Adverse Pregnancy Outcomes Group (n = 123)	*p* Value
	Biochemical Parameters		
Glucose (mg/dL)	77 (72–80)	78.5 (74–84)	*p* = 0.11
Insulin (µUI/mL)	5.8 (4.3–8.1)	6.3 (4.6–9.1)	*p* = 0.44
HOMA-IR index	1 (0.7–1.3)	1 (0.71–1.60)	*p* = 0.69
Triglycerides (mg/dL)	77 (67–95)	95 (74–123)	*p* = 0.001 *
Total Cholesterol (mg/dL)	171.8 ± 21.9	178.8 ± 31.4	*p* = 0.16
HDL Cholesterol (mg/dL)	65.1 ± 10.8	65.1 ± 10.9	*p* = 0.99
LDL Cholesterol (mg/dL)	56.1 ± 19	63.9 ± 20	*p* = 0.02 *
CRP (mg/L)	2.6 (1.5–4.7)	3.45 (2–8)	*p* = 0.04 *
Leukocyte count (×10^3^/μL)	8.29 ± 1.86	8.66 ± 1.97	*p* = 0.08
PlGF (pg/mL)	31 (25.5–42.7)	30.7 (23.1–44.3)	*p* = 0.95
sFlt-1 (pg/mL)	1579 (1257–2036)	1379 (1032–1666)	*p* = 0.005 *
sFlt1/PlGF ratio	48.4 (36.2–62.1)	43.2 (31.3–54.7)	*p* = 0.03 *
	Ultrasound Parameters		
SFT (mm)	17.1 (13.1–21.4)	18.9 (14.3–24.2)	*p* = 0.03 *
VFT (mm)	26.5 (19.5–35.9)	30 (23.4–45)	*p* = 0.001 *
Ratio of VFT-to-SFT	1.5 (1.1–2)	1.6 (1.2–2.1)	*p* = 0.24
Mean Uterine Arteries PI	1.58 (1.33–2.02)	1.58 (1.23–2)	*p* = 0.47

(*) Statistical significance *p* < 0.05.

**Table 3:** Relationships between mean ultrasound fat thickness measurements and biochemical and clinical parameters analyzed by Spearman correlation coefficient. (*) Statistical significance *p* < 0.05. CRP: C-reactive protein; SAP: systolic arterial pressure; DAP: diastolic arterial pressure; MAP: mean arterial pressure; BMI: Body Mass Index; PI: Pulsatility Index.

Variable	Visceral Fat Thickness	Subcutaneous Fat Thickness
Glucose	r = 0.11; *p* = 0.16	r = 0.08; *p* = 0.28
Insulin	r = 0.37; *p* < 0.001 *	r = 0.30; *p* < 0.001 *
HOMA-IR index	r = 0.33; *p* < 0.001 *	r = 0.34; *p* < 0.001 *
Triglycerides	r = 0.24; *p* = 0.02 *	r = 0.12; *p* = 0.11
Total Cholesterol	r = 0.08; *p* = 0.29	r = 0.10; *p* = 0.18
HDL Cholesterol	r = −0.09; *p* = 0.22	r = −0.03; *p* = 0.64
LDL Cholesterol	r = 0.11; *p* = 0.17	r = 0.12; *p* = 0.14
CRP	r = 0.47; *p* < 0.001 *	r = 0.28; *p* = 0.001 *
Leukocyte count	r = 0.09; *p* = 0.06	r = 0.07; *p* = 0.14
PlGF	r = −0.007; *p* = 0.92	r = -0.06; *p* = 0.45
sFlt-1	r = −0.06; *p* = 0.43	r = 0.04; *p* = 0.58
sFlt1/PlGF ratio	r = −0.05; *p* = 0.49	r = 0.09; *p* = 0.26
SAP	r = 0.16; *p* = 0.003 *	r = 0.32; *p* < 0.001 *
DAP	r = 0.20; *p* < 0.001 *	r = 0.30; *p* < 0.001 *
MAP	r = 0.20; *p* < 0.001 *	r = 0.27; *p* < 0.001 *
BMI	r = 0.57; *p* < 0.001 *	r = 0.60 *p* < 0.001 *
Mean Uterine Arteries PI	r = −0.03; *p* = 0.61	r = −0.16; *p* = 0.01 *

**Table 4:** Multivariate logistic regression analyses for the composite of adverse pregnancy outcomes, metabolic complications of pregnancy and vasculo-placental complications: adjusted Odds ratio (aOR) for maternal age, mean arterial pressure and smoking.

Variable	aOR	95% Confidence Interval	*p* Value
	Composite of Adverse Pregnancy Outcomes		
Subcutaneous fat thickness (mm)	0.99	0.96–1.03	*p* = 0.91
Visceral fat thickness (mm)	1.03	1.01–1.04	*p* < 0.001 *
Ratio of VFT-to-SFT	1.37	1.01–1.85	*p* = 0.04 *
BMI (Kg/m^2^)	1.03	0.98–1.08	*p* = 0.2
	Metabolic Complications		
Subcutaneous fat thickness (mm)	1.04	1.004–1.08	*p* = 0.03 *
Visceral fat thickness (mm)	1.07	1.04–1.09	*p* < 0.001 *
Ratio of VFT-to-SFT	2.09	1.35–3.25	*p* = 0.001 *
BMI (Kg/m^2^)	1.11	1.03–1.19	*p* = 0.03 *
	Placental Vascular Dysfunction Complications		
Subcutaneous fat thickness (mm)	0.95	0.91–1.004	*p* = 0.07
Visceral fat thickness (mm)	0.98	0.96–1.005	*p* = 0.13
Ratio of VFT-to-SFT	1.11	0.76–1.62	*p* = 0.56
BMI (Kg/m^2^)	0.97	0.91–1.03	*p* = 0.36

(*) Statistical significance *p* < 0.05.

## Data Availability

Not applicable.
